# The regulation of triglyceride storage by *Acsx4* and *Acsx5* in *Drosophila* fat tissue

**DOI:** 10.17912/micropub.biology.001430

**Published:** 2025-02-01

**Authors:** Erick Astacio, Justin R. DiAngelo

**Affiliations:** 1 Division of Science, Penn State Berks, Reading, PA

## Abstract

The production of energy is one of the most fundamental requirements for organismal survival. Decreasing expression of *Drosophila* *9G8* , an mRNA splicing protein, specifically in adipose tissue results in triglyceride accumulation. Decreasing *9G8* in adipose also results in upregulation of the acyl-CoA synthetases *Acsx4* and *Acsx5* ; however, the functions of these genes in regulating lipid metabolism is not fully understood. Here, we decreased *Acsx4* and *Acsx5* in fly adipose tissue and this resulted in high triglycerides. This suggests that these genes regulate lipid breakdown, and their upregulation is perhaps compensating for the triglyceride accumulation observed when *9G8* levels are decreased.

**Figure 1. Decreasing expression of Acsx4 and Acsx5 in the female fat body results in an increase of triglycerides:**
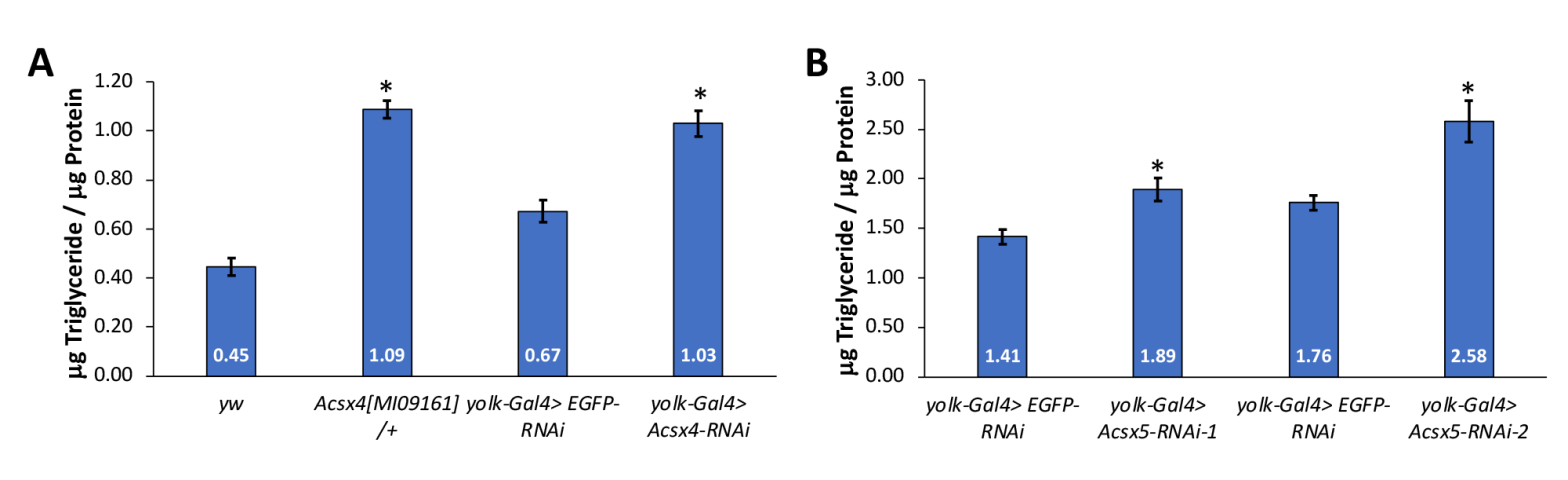
(A) Triglycerides were measured in *Acsx4 ^[MI09161]^ /+* (n=11 from 4 independent crosses) and *yolk-Gal4>Acsx4-RNAi* (n=45 from 5 independent crosses) females and compared to *yw* (n=16 from 6 independent crosses) and *yolk-Gal4>EGFP-RNAi* (n=51 from 5 independent crosses) female controls, respectively. (B) Triglycerides were measured in *yolk-Gal4>Acsx5-RNAi-1* (n=35 from 3 independent crosses) and *yolk-Gal4>Acsx5-RNAi-2* (n=32 from 3 independent crosses) females and compared to their respective *yolk-Gal4>EGFP-RNAi* control females (n=32-35 from 3 independent crosses). Triglyceride was quantified and divided by protein concentration. Bars indicate average levels of triglyceride/protein +/- standard error. Unpaired, two-sample t-tests were conducted and a *P<0.05 indicates statistical significance.

## Description

Alteration of the pathways that produce energy such as the breakdown of carbohydrates and fats can result in chronic disorders such as obesity and type 2 diabetes (Hotamisligil, 2006) . These disorders have been classified as a major global crisis due to the comorbidities associated with them, so a better understanding of the metabolic pathways that are altered in obesity and type 2 diabetes will hopefully help with the prevention of these diseases (Verma and Hussain, 2017) . Excess storage of triglycerides is observed in both obesity and type 2 diabetes; however, all the genes responsible for the regulation of nutrients stored as triglycerides is not known.

To identify and better understand the genes important for triglyceride storage, the fruit fly, *Drosophila melanogaster* , has been selected as a useful model organism to meet this objective. Flies are an excellent model system to study fat metabolism since they share similar genes to humans and have analogous organs to humans such as the fat body which functions like human liver and adipose tissue (Musselman and Kuhnlein, 2018) . To identify novel genes responsible for lipid storage, genome-wide RNA interference (RNAi) screening was performed in *Drosophila* cells, and many different types of genes were identified (Guo et al., 2008; Beller et al., 2008) . Splicing factors, proteins involved in the processing of mRNAs to generate mature transcripts, were among the genes identified in these screens (in each of the two RNAi screens described above, approximately 7% of the genes that resulted in fewer/smaller lipid droplets when knocked down were involved in mRNA splicing) and our lab has characterized several splicing factors that act in fat tissue to regulate triglyceride storage. Approximately 10% of the splicing factors from the Guo and Beller screens were SR proteins, a family of proteins that function to identify intron/exon borders (Long and Caceres, 2009) , and two of the eight SR proteins in the *Drosophila* genome were identified in these screens (Guo et al. 2008; Beller et al., 2008) . We have specifically focused on SR proteins in regulating metabolic homeostasis in *Drosophila* . For example, when the expression of one SR protein, *9G8* , was decreased specifically in fat tissue, the flies displayed a high triglyceride storage phenotype (Gingras et al., 2014) . To identify the genes regulated by 9G8 to result in this accumulation of triglycerides, RNA sequencing was performed on fat tissue with *9G8* levels decreased (Weidman et al., 2022) . Interestingly, two novel genes predicted to be acyl-CoA synthetases (ACS) (named *CG4830* and *CG11453* ) were identified as being upregulated when *9G8* expression was decreased (Weidman et al., 2022) . However, whether these ACS genes are responsible for promoting triglyceride storage when *9G8* is decreased is not known.

Both *CG11453* and *CG4830* are members of a group of ACS genes that are found specifically in worms and flies (Watkins et al, 2007) . Since the substrates of these ACS enzymes are not characterized, this group of ACS genes has been referred to as *Acsx* (Watkins et al, 2007) and *CG11453* has already been named *Acsx4* . Since *CG4830* has not been characterized, but is a member of this *Acsx* gene group, we have decided to name this gene *Acsx5* . ACS enzymes are responsible for adding CoA groups to fatty acids and these fatty acids can either be targeted for breakdown via beta-oxidation or they can be esterified with glycerol to make triglycerides (Watkins, 1997) . However, whether *Acsx4* and *Acsx5* function in *Drosophila* to promote fatty acid oxidation or triglyceride esterification is not known.

To characterize the role of *Acsx4* in regulating lipid metabolism in *Drosophila* , triglycerides were measured in flies heterozygous for a transposon insertion in *Acsx4 (Acsx4 ^[MI09161]^ /+)* . In these *Acsx4* mutant flies, an increase in triglyceride was observed (Fig 1A). To determine if the increased triglycerides in *Acsx4* mutants results from *Acsx4* function in the fat body, RNAi towards *Acsx4* was induced specifically in the adult female fat body *.* The knockdown of *Acsx4* via RNAi in adipose tissue showed similar increases in triglyceride observed in the *Acsx4* mutants (Fig 1A). To assess the function of *Acsx5* in regulating lipid storage, RNAi towards *Acsx5* was induced specifically in the fat body using two independent RNAi lines. When *Acsx5* was decreased using both RNAi lines, triglyceride accumulated, similar to the phenotypes observed with *Acsx4* (Fig 1B). Together, these data suggest that both *Acsx4* and *Acsx5* limit lipid storage, perhaps by promoting the addition of CoA groups onto fatty acids to target them for beta-oxidation in the mitochondria. In addition, the upregulation of these genes in adipose-specific *9G8-RNAi* flies is probably compensating for the increased triglycerides observed in these flies as the fold increase in triglycerides in fat body-specific *9G8-RNAi* flies is much higher than the fold increases in triglycerides observed in fat body-specific knockdown of *Ascx4* or *Acsx5* (Gingras et al., 2014; Fig 1).

Two additional acyl-CoA synthetases, *pudgy* and *bubblegum* , have been identified in *Drosophila* to regulate triglyceride metabolism. Overexpressing *pudgy* in the fat body *in vivo* results in blunted organismal fat levels, while decreasing *pudgy* increases triglyceride storage (Xu et al., 2012) . Another acyl-CoA synthetase in flies, *bubblegum* , was found to exhibit elevated levels of very long chain fatty acids when its expression was decreased (Min and Benzer, 1999) . These phenotypes in previously characterized *Drosophila* acyl-CoA synthetases are consistent with the phenotype observed when *Acsx4* and *Acsx5* were knocked in the fat body of female flies, suggesting that *Drosophila* have multiple functional acyl-CoA synthetases that regulate lipid breakdown. Additional experimentation is needed to determine the regulation of these enzymes in different tissues and throughout different stages in *Drosophila* development to regulate metabolic homeostasis.

Acyl-CoA synthetases are also well conserved in mammals. Five subfamilies of mammalian acyl CoA synthetases have been identified in which the length of the carbon chain of the fatty acid species defines the substrate specificity for these enzymes (Soupene and Kuypers, 2008) . The function of one of these families, long-chain acyl-CoA synthetase-1 (ACSL1), has been studied in both mouse and rat adipose tissue. When *ACSL1* was knocked out in the adipose tissue of mice, triglycerides accumulated, and a 50-90% lower beta oxidation rate was observed when compared to the controls (Ellis et al., 2010) . This suggests a role for *ACSL1* in regulating lipid breakdown which is like what we see with *Acsx4* and *Acsx5* in *Drosophila* . Moreover, *ACSL1* mRNA and protein levels have also been shown to be regulated by fasting and refeeding in rats (Mashek et al., 2006) , suggesting that perhaps diet regulates *Acsx4* and *Acsx5* expression in *Drosophila* fat tissue. Future experiments are necessary to address any regulation of *Acsx4* and *Acsx5* by different dietary conditions in *Drosophila* .

## Methods

**Fly genetics**

Experimental crosses of virgin GAL4 females with UAS males were performed at 25°C in a 12h:12h light: dark cycle. *Acsx4 ^[MI09161]^ /TM3, Sb[1] Ser[1]* flies were crossed to *yw* background controls to generate *Acsx4 ^[MI09161]^ /+* flies used in this study. Flies were grown on sugar-yeast-cornmeal food (9g *Drosophila* agar (Genesee Scientific), 100mL Karo Lite Corn Syrup, 65g cornmeal, 40g sucrose, and 25g whole yeast in 1.25L water). Flybase was used to find background information and fly stocks available for *Acsx4* and *Acsx5* (Öztürk-Çolak et al., 2024).

**Triglyceride and Protein Measurements**

Throughout this study one-week old female flies were used; females were mated and aged with male flies. Two whole flies were homogenized in lysis buffer (140 mM NaCl, 50 mM Tris-HCl, pH 7.5, 0.1% Triton-X, and 1X protease inhibitor (ThermoFisher, Waltham, MA, USA)). Triglyceride and protein were measured using the Infinity Triglyceride Reagent kit (ThermoFisher, Waltham, MA, USA)), and Pierce BCA Protein Assay kit (ThermoFisher, Waltham, MA, USA), respectively, according to manufacturer's instructions and as previously described (Bennick et al., 2019) .

**Statistics**

The mean triglyceride/protein results were analyzed comparing values between *Acsx4 ^[MI09161]^ /+* mutants and *yw* controls, and the *yolk-Gal4>Acsx4-RNAi* , and *yolk-Gal4>Acsx5-RNAi* flies and respective *yolk-Gal4>EGFP-RNAi* control flies via an unpaired, two-sample t-test as calculated in Microsoft Excel. A p<0.05 was used to determine statistical significance.

## Reagents

**Table:** 

**Fly strain**	**Genotype**	**Stock Number**
*yw*	*y[1] w[1]*	BL#1495
*Acsx4[MI09161}/ TM3, Sb[1] Ser[1]*	y[1] w[*]; Mi{y[+mDint2]=MIC} Acsx4[MI09161] /TM3, Sb[1] Ser[1]	BL#51390
*UAS-EGFP-RNAi*	y[1] sc[*] v[1] sev[21]; P{y[+t7.7] v[+t1.8]=VALIUM20-EGFP.RNAi.1}attP40	BL#41555
*UAS-Acsx4-RNAi*	y[1] sc[*] v[1] sev[21]; P{y[+t7.7] v[+t1.8]=TRiP.HMC03954}attP40	BL#55267
*UAS-Acsx5-RNAi-1*	y[1] sc[*] v[1] sev[21]; P{y[+t7.7] v[+t1.8]=TRiP.HMC04535}attP40	BL#57155
*UAS-Acsx5-RNAi-2*	*y[1] v[1]; P{y[+t7.7] v[+t1.8]=TRiP.HMJ24152}attP40*	BL#62912
*yolk-Gal4*	y[1] w[*]; P{w[+mC]=yolk-GAL4}2	BL#58814
